# Targeted Agents in the Treatment of Indolent B-Cell Non-Hodgkin Lymphomas

**DOI:** 10.3390/cancers14051276

**Published:** 2022-03-01

**Authors:** Adrian Minson, Constantine Tam, Michael Dickinson, John F. Seymour

**Affiliations:** 1 Peter MacCallum Cancer Centre & Royal Melbourne Hospital, Melbourne, VIC 3000, Australia; constantine.tam@petermac.org (C.T.); michael.dickinson@petermac.org (M.D.); john.seymour@petermac.org (J.F.S.); 2 Sir Peter MacCallum Department of Oncology, University of Melbourne, Parkville, VIC 3010, Australia

**Keywords:** non-Hodgkin lymphoma, targeted therapy, personalised medicine, immunotherapy

## Abstract

**Simple Summary:**

New therapies for lymphoma continue to be developed at a rapid rate, including for the indolent lymphomas. While traditional cytotoxic chemotherapy remains an effective treatment option, many new treatments target specific aspects of cancer cells such as surface proteins or signaling pathways with the aim of providing a more personalized and tailored approach. By doing so, targeted agents can interrupt cancer cell growth and development, make cancer cells more susceptible to other treatments, or direct the immune system to recognize and destroy cancer cells. This review highlights and discusses the currently available and emerging targeted therapies that apply to follicular lymphoma, lymphoplasmacytic lymphoma/Waldenstrom macroglublinaemia and marginal zone lymphoma.

**Abstract:**

Targeted therapies continue to change the landscape of lymphoma treatment, resulting in improved therapy options and patient outcomes. Numerous agents are now approved for use in the indolent lymphomas and many others under development demonstrate significant promise. In this article, we review the landscape of targeted agents that apply to the indolent lymphomas, predominantly follicular lymphoma, lymphoplasmacytic lymphoma/Waldenstrom macroglobulinaemia and marginal zone lymphoma. The review covers small molecule inhibitors, immunomodulators and targeted immunotherapies, as well as presenting emerging and promising combination therapies.

## 1. Introduction/Background

The landscape of targeted therapies in indolent lymphoma continues to expand rapidly and advances in this arena have resulted in significant improvements in patient outcomes. The range of these agents spans multiple domains and mechanisms of action, and includes monoclonal antibodies, small molecule inhibitors, protein degraders and cellular immunotherapies. A host of agents are now licensed or in late clinical development and even more have shown early promise. 

In this review, we focus on the landscape of targeted agents in indolent lymphoma, particularly follicular lymphoma (FL), lymphoplasmacytic lymphoma/Waldenström macroglobulinaemia (LPL/WM) and marginal zone lymphoma (MZL). Numerous excellent reviews of targeted agents can already be found covering chronic lymphocytic leukaemia (CLL) [1,2,3], mantle cell lymphoma (MCL) [4,5] and diffuse large B-cell lymphoma (DLBCL) [6,7].

## 2. Review of Approved Small Molecule Inhibitors

### 2.1. B-Cell Receptor Pathway Agents

#### 2.1.1. BTK

Bruton tyrosine kinase (BTK) is a multi-domain TEC-family kinase involved in key pathways of B-cell development and homing [8]. BTK can be activated through a variety of mechanisms, including chemokine signalling, antigen-dependent B-cell receptor (BCR) stimulation and antigen-independent pathways (e.g., Toll-like receptor) [9,10]. Activation leads to phosphorylation of signalling molecules, including phospholipase Cγ (PLCγ), extracellular signal-regulated protein kinase 1/2 (ERK1/2), recruitment of the CARD11/MALT1/BCL10 complex and ultimately activation of NF-kB, AP1 and IRF3 with associated target gene transcription and cellular proliferation [8,9]. The centrality of BTK activity has been implicated in the development and survival of a number of B-cell malignancies, particularly those demonstrating BCR and/or NF-kB pathway dependency [8,9,11,12].

All currently approved BTK inhibitors covalently bind in an irreversible fashion to the Cys481 residue in the catalytic ATP-binding domain of BTK, thereby interrupting physiologic function. In addition to its BTK affinity, the first-in-class agent ibrutinib exhibits a relatively broad spectrum of kinase inhibition at clinically relevant concentrations, including ITK, TEC and the EGF receptor leading to potential off-target effects [13,14]. While this may account for some observed toxicities, these changes may also play a role in therapeutic activity. Inhibition of ITK, for example, is associated with broader immunologic changes, including skewing towards a Th1 CD4+ phenotype and improved T-cell expansion and fitness [15,16,17]. Ibrutinib first came to prominence in studies of CLL, where it was demonstrated to induce high rates of overall response in both previously treated and untreated patients [18,19]. Ibrutinib activity has since been extended to indolent B-cell lymphomas, as detailed in Table 1. Notably, ibrutinib has demonstrated only modest single-agent activity in R/R FL, with ORR in a total of 150 patients in two studies of between 21–37.5% and median PFS of 4.6–14 months [20,21]. CARD11 mutations predicted for absence of response as anticipated through downstream bypassing of BTK-dependency, but even among CARD 11 wild-type patients the median PFS was approximately 17 months [21]. Activity in combination in the frontline setting continues to be evaluated (NCT02947347, NCT04450173 and NCT01829568) [22]. In contrast to FL, efficacy in MZL and WM is more consistently demonstrated, including in both phase 2 and randomised phase 3 studies (Table 1).

A number of second-generation irreversible covalent agents have been designed to provide greater selectivity for BTK, with two agents, acalabrutinib and zanubrutinib, licensed for use in CLL and MCL and newer agents undergoing early phase studies (Table 1). Although targeting the same cysteine residue as ibrutinib, physiochemical properties reduce the ability of these agents to interact with other kinases, thereby minimising toxicity while preserving efficacy [31,32]. Three phase 3 randomised controlled trials of acalabrutinib or zanubritinib, compared to ibrutinib in various lymphoma subtypes, have each demonstrated an improved safety profile of the second generation agents, and, particularly, lower rates of atrial fibrillation, which was observed in 2–2.5% with zanubrutinib and 9% acalabrutinib, compared to approximately 15% with ibrutinib [28,33,34]. Acalabrutinib has predominantly been developed in CLL and MCL, and is FDA approved for both indications [35]. A phase 2 study in treatment naïve and relapsed refractory WM showed high levels of efficacy in both settings, including ORR of 93% and PFS of 82% in relapsed disease at a median follow up of 27.4 months [27]. Zanubrutinib is FDA approved in R/R MCL on the basis of two early phase studies, with a phase 2 single-arm study of 86 patients with a median of two lines of prior treatment demonstrating a CR rate of 68.6% and median DOR of 19.5 months [36,37]. There is accumulating evidence of activity of zanubrutinib in both MZL and WM, including a phase 3 study comparing zanubrutinib to ibrutinib in the latter disease showing equivalent overall response rate, depth of response and a favourable adverse event profile, including a lower rate of drug discontinuation [24,28]. Two additional covalent BTK inhibitors, tirabrutinib and oralebrutinib, demonstrate promise in WM, both demonstrating high response rates and significant durability in R/R disease with median PFS not reached at follow up of 25 and 11 months, respectively [29,30].

Despite the significant success of covalent BTK inhibitors, each is limited by the possibility of primary or acquired resistance [10]. The most direct mechanism of resistance is the mutation of BTK itself, either within (e.g., C481S) or outside the active kinase domain, preventing inhibitor binding or bypassing their activity [38]. However, multiple other mechanisms have been elucidated, including activation of downstream molecules, over-activity of alternative pathways of NF-kB generation and increased expression of anti-apoptosis proteins [10]. Patterns of resistance are not necessarily shared between disease types; therefore, it is important to understand these differences to drive rational selection of subsequent or combination therapies [39].

One approach to overcoming resistance has been to focus on alternative BTK binding strategies. The development of highly potent, selective, non-covalent, reversible inhibitors have proven effective in patients with disease progressing on covalent BTK inhibitors and those harbouring a *BTK*C481 mutation [40,41]. At least four such agents have progressed through various phases of development, with pirtobrutinib (LOXO-305), vecabrutinib (SNS-062) and nemtabrutinib (MK-1026; formerly ARQ-531) the most advanced [39,42,43]. The phase 1/2 BRUIN study tested pirtobrutinib monotherapy in 323 patients with relapsed or refractory B-NHL, the majority of whom had prior exposure to a BTK inhibitor, with an overall response of 68% in WM (*n* = 26), 50% in FL (n = 12) and 22% in MZL (n = 9) observed. The most common toxicity was fatigue in 20%, with other side effects observed less frequently than with covalent agents [40]. In contrast, vecabrutinib displayed only minor clinical benefit despite satisfactory pharmacokinetics and excellent tolerability, which may partially be explained by the high percentage of patients in the phase 1b study with adverse prognostic features, including *TP53* aberrancy (73.7%) and/or *BTK*C481 mutations (44.7%) [43]. In addition to BTK binding, novel agents causing BTK degradation are also in development, the key advantages being activity independent of the presence of *BTK* C481mut and the ability to degrade active, phosphorylated BTK [44].

Combination therapies are an additional strategy being explored to augment the efficacy of first and subsequent generation BTK inhibitors [9]. Synergism with the antitumor effects as well as the broader immunologic properties of BTK inhibitors provide rationale for use with both targeted and immunotherapeutic agents. For instance, the ITK effects of ibrutinib on T-cell fitness may potentiate T-cell based treatments, including CAR T-cells [45,46,47] and bispecific T-cell recruiting antibodies [48]. Other examples of combination studies are presented throughout the text below. 

#### 2.1.2. PI3K/AKT/mTOR Pathway

PI3K is a lipid kinase present in most tissue types. It exists in four isoforms and is involved in controlling numerous cellular processes, including metabolism, motility, growth and proliferation [49]. In lymphocytes, the predominantly expressed isoform is PI3Kδ, which acts downstream of the BCR and pre-B cell receptor, playing a crucial signalling role in B cell survival through the AKT and mTOR pathway [12]. Agents targeting the PI3K/AKT/mTOR pathway have been in development for more than two decades and have been approved for use in a number of cancers. 

In indolent lymphoma, the key class is the PI3K inhibitors. Four FDA approved agents have demonstrated efficacy in indolent B-NHL (Table 2). Idelalisib, duvelisib and umbralisib all act on the delta isoform, whereas copanlisib acts primarily on the gamma and alpha isoforms, contributing to its distinct side effect profile. Although cross-trial comparison is fraught, the PI3K inhibitors have broadly similar overall response rates of between 40–60% in FL, with comparable durability of responses, including median progression free survival between 9–12 months [50,51,52,53,54]. Umbralisib is the sole agent FDA approved for use in marginal zone lymphoma, where overall response rates were similar to that observed in follicular lymphoma. Of note, durability of response was prolonged, with two thirds of responses on-going beyond 2 years, and the adverse event profile was comparable [54]. 

Toxicity has been a major impediment to the clinical uptake of PI3K inhibitors, in part due to the ubiquity of PI3K isoforms in other tissue types [49]. Key adverse events include gastrointestinal upset, hepatotoxicity, pneumonitis and infections (Table 2). Immune mediated phenomena, such as colitis, hepatitis and pneumonitis are thought to be mediated via T-cell effects predominantly through the delta isoform and are therefore less prominent with copanlisib [55] and the newer generation umbralisib [54]. Toxicities can occur early, such as diarrhoea and hepatotoxicity (mostly within the first 12 weeks), while others can be late events, such as pneumonitis occurring up to 15 months after treatment initiation [56]. Rates of these toxicities vary according to treatment timing (more prominent in frontline vs. relapsed setting), disease subtype and whether used in combination [57]. Regardless, treatment discontinuation due to toxicity remains consistently high (between 15–30%) and the leading cause of treatment withdrawal in most clinical studies (Table 2). 

Resistance mechanisms are still being elucidated, but it is clear that reactivation of PI3K signalling through alternate pathways emerges as a central theme [58]. This forms the basis for the development of combination inhibitors, such as those targeting both PI3K/AKT or PI3K/mTOR, with a number of agents undergoing investigation [58,59,60]. In addition, combining PI3K with other immunotherapies or chemotherapy has been explored, although this again has been hampered by excessive toxicity. For instance, the combination of idelalisib plus lenalidomide and rituximab in FL and MCL resulted in >70% treatment discontinuation [61]. A phase 1 study of idelalisib with rituximab and/or bendamustine demonstrated high response rates in heavily treated R/R B-NHL, although later phase trials showed an increase in toxicity not evident in the original phase 1 [62]. Other combination studies include idelalisib in combination with obinutuzumab in FL (NCT03890289) or WM [63], duvelisib with acalabrutinib in B-NHL (NCT04836832), umbralisib with rituximab in FL/MCL (NCT03919175), with ublituximab in FL (NCT03828448) or with ublituximab-CHOP in MCL (NCT04692155). The potential for synergy with venetoclax provides a strong rationale for assessing this combination, given pre-clinical studies showing the ability of PI3K inhibitors to sensitize FL cells to BCL2 inhibition through microenvironmental effects [64]. 

### 2.2. BCL2

BCL2 is a key pro-survival protein acting within the apoptotic pathway. Deregulation of BCL2 is observed in many haematological malignancies, including indolent lymphomas, and translocation of BCL2 with the IgH promoter regions is the hallmark genetic change characterising FL. Altered expression of BCL2 inhibits programmed cell death by sequestering BAK and BAX proteins, perturbing the balance between pro-survival and pro-apoptotic signals [65,66]. In cancerous cells that utilise aberrant BCL2 to abrogate pro-death signals, inhibition of BCL2 can lead to rapid triggering of apoptosis [65]. The main mechanism to achieve this is through mimicking the activity of BH3-only peptides, such as Bad, which binds to BCL2, releasing previously quenched pro-apoptotic protein, such as Bim, resulting in unopposed activity of BAK and BAX [67].

Venetoclax is a potent BH3-mimetic that has been approved for use in CLL. Single agent venetoclax, or venetoclax combined with an anti-CD20 antibody, has been explored in FL, WM and MZL in the relapsed/refractory setting (Table 3). Overall modest monotherapy activity has been demonstrated in FL (ORR 35–38%) with no clear additional benefit from venetoclax demonstrated when combined with BR chemotherapy [68]. Early results in MZL and WM are more encouraging. In a phase 2 study of venetoclax monotherapy in 32 patients with WM, an ORR of 84% was observed with a median PFS of 30 months, including significant activity after BTKi failure or in the presence of *CXCR4* mutations [69].

Mechanisms of resistance have been elucidated most clearly in the setting of CLL, where mutations in BCL2, overexpression of pro-survival proteins MCL1 and BCLxL and aberrancy in other essential pathways, such as growth signalling, cell cycling and epigenetic regulation have been demonstrated [66]. Combination therapy with a second mechanism of action has been attempted to augment the anti-lymphoma effects while avoiding the development of resistant clones. Most prominently, the combination of BTKi and BCL2 has been effective in CLL, MCL and MZL and is being tested in R/R FL (NCT02956382) [72,73,74,75]. In a phase 2 study of 12 patients with R/R MZL, an ORR of 58% and CRR of 25% was observed at week 16 [74]. Adverse events included atrial fibrillation (17%), grade 3/4 neutropenia (17%) and thrombocytopenia (8%). Early results from a phase 1 study of zanubritinib in combination with BGB-11417 (a selective BCL2 inhibitor) has demonstrated excellent tolerability with no grade ≥3 events, although efficacy has not yet been established due to short follow up [76]. Phase 3 studies are currently investigating this combination in relapse and upfront therapy, predominantly in CLL and MCL [77,78].

Other combination studies are also active, including in the frontline setting. Preliminary results for the combination of venetoclax/obinutuzumab/polatuzumab in FL [79] and venetoclax/obinutuzumab/lenalidomide in MZL/FL [80] demonstrate promising activity and studies combining atezolizumab (NCT03276468) and copanlisib (NCT03886649) are on-going. The triplet combination of venetoclax, obinutuzumab, lenalidomide is also being explored in the upfront LEVERAGE study in FL (NCT03980171). 

### 2.3. EZH2

Epigenetic deregulation is an important mechanism contributing to lymphomagenesis. DNA methylation, a cornerstone of the epigenetic program, is critical for the precise regulation of gene expression involved in normal lymphocyte development [81]. An enhancer of zeste, homolog 2 (EZH2) is a histone methyltransferase that catalyses the methylation of H3K27 resulting in controlled repression of gene transcription. Interestingly, the contribution of EZH2 aberrancy to lymphomagenesis differs between lymphocyte subsets; overexpression is tumorigenic in B-cells, whereas under-expression has this same effect in T-cells [82]. EZH2 is particularly active in germinal centre B-cells, where this repressive function regulates genes involved in cell cycling, B-cell differentiation and maturation [82,83]. Abnormal upregulation of activity in cells derived from this origin can therefore lead to excess proliferation and differentiation block. EZH2 is recurrently mutated in indolent lymphoma, most prominently in FL, where mutations have been found in 28% of cases [84]. 

Tazemetostat interrupts EZH2 function by competitively inhibiting the substrate used to methylate H3K27, and is therefore active against both mutant and wildtype EZH2 [85]. This agent has received FDA approval on the basis of a phase 2 multi-cohort study of monotherapy in R/R FL after at least two prior therapies [86]. Ninety-nine patients were enrolled at a median age of 61 years, 54 with wild-type *EZH2* and 45 harbouring an *EZH2* mutation. PFS and DOR were similar between mutated and unmutated groups (13.8m, 11.1 m, 10.9 m and 13.0 m, respectively), although complete responses (13% vs. 4%) and the achievement of any degree of cytoreduction (69% vs. 35%) were greater amongst those carrying mutations. Treatment at a dose of 800 mg twice daily was well tolerated with a low rate of serious adverse events. While gain-of-function *EZH2* mutations were the genetic lesion most significantly associated with response to treatment, additional mutations have also been identified as having a positive effect on PFS (*ARID1B*, *TP53* and *HIST1H1C*) and OS (*FOXP1*) [87]. The presence of these mutations in other indolent lymphomas, such as *ARID1B* in WM, may indicated a future role of tazemetostat in these entities [88]. Combination studies in FL of tazemetostat with rituximab [89], lenalidomide [90] or both (NCT04224493) are on-going. Additional EZH2 inhibitors are also being explored in haematologic malignancies, including phase 1 studies of SHR2554 (NCT03603951), PF-06821497 (NCT03460977) and GSK2816126 [91], demonstrating modest efficacy.

### 2.4. Upcoming Targeted Agents

#### 2.4.1. XPO1

XPO1 is a member of the karyopherin family of nuclear transporters and mediates the export of nuclear biomolecules [92]. Protein cargos transported by XPO1 include known tumour-suppressors, such as p53 and factors regulating cellular growth, such as MYC and MDM2 [93]. Overexpression of XPO1 can therefore lead to the improper localisation of these important mediators and is a feature of many malignancies. While inhibition will affect healthy cells, cancerous cells reliant on XPO1 activity may be uniquely susceptible [93].

Selinexor is a selective inhibitor of the XPO1-mediated nuclear export that is approved for use in R/R multiple myeloma and DLBCL. In the latter, a phase 2B study of 127 patients demonstrated an overall response rate of 28%, including 12% CR, and a median PFS of 2.6 months in a heavily pre-treated population (median 2–5 lines of therapy) [94]. Serious adverse events were common (48% of patients), including grade 3–4 thrombocytopenia in 46%, with 70% of patients requiring dose interruption or reduction. Limited data exist on the use of selinexor in indolent lymphomas. A phase 1 portion of a study of selinexor in combination with R-CHOP included two patients with FL and four with transformed FL, demonstrating a high complete response rate in both groups [95]. A number of studies of selinexor including participants with indolent lymphomas are on-going (NCT03955783, NCT02741388 and NCT04640779). 

#### 2.4.2. MCL-1

There are a number of pro-survival proteins of the BCL-2 family that act similarly to BCL-2 within the apoptotic pathway, including BCL-xL, MCL-1 and BLF1 [66]. Distinct patterns of dependence on these pro-survival proteins has been demonstrated in specific haematological malignancies [96]. For instance, while BCL-2 predominates in CLL, MCL-1 plays a more significant role in myeloid disorders. Even amongst B-cells there is a spectrum of dependency; MCL-1 is important for B-cell germinal centre formation and plasma cell survival, whereas BCL-2 is essential for naïve B cells [97]. BH3-mimetics can be tailored for greater specificity towards one or more of the pro-survival mediators, and there are now numerous MCL-1 inhibitors in clinical development [66,97]. 

To date, there are limited clinical efficacy and toxicity data regarding MCL-1 inhibitors in lymphoid malignancies. AMG-176 is an intravenous MCL-1 inhibitor that was tested in 26 patients with relapsed-refractory myeloma, demonstrating acceptable haematologic and gastrointestinal toxicity and modest rates of response [98]. MCL-1 overexpression is a common finding in B-cell lymphomas, including as a resistance factor to venetoclax [99,100]. However, the degree of MCL-1 dependence has generally been correlated with more aggressive lymphoma subtypes [101] and, therefore, while there is a rationale to explore MCL-1 inhibitors in indolent lymphomas, the anticipated efficacy may be lower. 

#### 2.4.3. MDM2

MDM2 is an E3 ubiquitin ligase that targets p53 for degradation via a negative feedback loop [102]. P53 is an essential transcription factor playing a tumour suppressor role by activating target genes that ultimately lead to apoptosis. Dysregulation of p53 is a common feature of lymphoid malignancies and is almost universally considered a poor prognostic feature associated with treatment resistance, particularly to cytotoxic chemotherapy [103]. MDM2 upregulation is a mechanism exploited by some tumour types, leading to excess degradation of p53 and a reduction in normal apoptotic homeostasis [102]. Inhibition of MDM2 therefore has the potential to re-sensitise malignant cells to normal cell-death signals and may synergise with other therapeutic agents targeting the apoptotic pathway. 

Idasanutlin is one of a number of MDM inhibitors with emerging clinical data in haematologic disorders, including AML, myeloproliferative neoplasms and lymphoid malignancies [102]. Early dose finding studies of this agent were limited by gastrointestinal toxicity resulting in a 5 out of 28 days dosing schedule [104]. Phase I/II trials testing idasanutlin in combination with obinutuzumab [105] or with obinutuzumab/rituximab and venetoclax [106] in FL and DLBCL demonstrated generally tolerable safety profiles. However, further development was halted, partly due to limited response rates using these combinations [106]. Indeed, while MDM2 oncoprotein overexpression appears common in DLBCL irrespective of *TP53* status, overexpression may not be as frequent in indolent lymphoma [107], suggesting that targeting this part of the MDM2-P53 signalling pathway may be insufficient in the indolent lymphoma context. Nevertheless, a number of other MDM2 inhibiting agents continue to be developed, including in lymphoma (NCT04502394, NCT02935907 and NCT02264613). 

#### 2.4.4. BET

BET family proteins are part of a larger group of bromodomain proteins that are involved in epigenetic regulation through histone modification. BET proteins bind to acetylated lysine residues on histone tails and promote recruitment and stabilisation of transcriptional complexes, particularly to super-enhancer regions, leading to expression of oncogenes, such as *c-MYC* and *NF-kB* [108,109]. Malignant cells utilising oncogenes associated with super-enhancers may be especially sensitive to BET inhibition [110]. In addition, BET inhibitors may also work through stimulating BIM-mediated intrinsic apoptosis [111]. 

To date, small numbers of patients with indolent lymphoma have been included in early-phase trials of BET inhibitors. Efficacy in this patient group has typically been lower than in patients with aggressive histology diseases [110,112,113,114], and, for this reason, clinical development has focused on DLBCL. In a phase 1 study of BETi RO6870810 monotherapy in patients with DLBCL, objective responses were observed in 11% [115]. A combination study with venetoclax and rituximab in R/R DLBCL demonstrated ORR of 38.5%, including 20.5% CR [116]. However, despite the promising rationale, there was no convincing synergy between concurrent BET and BCL2 inhibition. 

#### 2.4.5. SYK

Spleen tyrosine kinase (SYK) is an important cytosolic component of the BCR signalling pathway, contributing to initiation and amplification. Conformational change of CD79A and CD79B after BCR activation results in the phosphorylation of SYK, which in turn interacts with downstream molecules, most prominently BTK and PI3Kδ [11,117]. Inhibition of SYK therefore follows a similar rationale to the BTK and PI3K inhibitors.

Two main SYK inhibitors have entered clinical studies in indolent lymphomas: fostamatinib and entospletinib. The former was tested in a phase 1/2 study in patients with R/R B-NHL, demonstrating ORR of 10% in FL, 55% in CLL/SLL and no responses in three patients with MZL or MALT lymphoma [118]. Dose-limiting toxicities included GI toxicity, neutropenia and thrombocytopenia. Entospletinib is a second-generation selective inhibitor with a more favourable toxicity profile that demonstrated similar efficacy. In a cohort of 114 patients with indolent lymphoma consisting of FL, LPL, MZL and MCL, the ORRs were 17%, 35%, 12% and 18% and PFS 5.7 m, 10.9 m, 5.5 m and 5.6 m, respectively [119]. 

This modest single-agent efficacy suggests that SYK inhibition alone is insufficient as a strategy in indolent lymphoma. The position of SYK at the commencement of the BCR cascade may provide multiple opportunities for malignant cells to bypass this effect downstream, especially though non-antigen-dependent signalling pathways. Therefore, combination treatments are in development, including with inhibitors of JAK1/3 (cerdulatinib), FLT-3 (TAK-659), BCL-2 (NCT03357627) or cytotoxic chemotherapy (NCT02954406). 

#### 2.4.6. MALT1

Mucosa-associated lymphoid tissue lymphoma translocation protein 1 (MALT1) is a unique paracaspase and key component of the CARD11/MALT1/BCL10 complex that mediates NF-κB signalling [120,121]. When dysregulated, MALT1 acts as a proto-oncogene and is known to contribute to tumorigenesis in lymphoid malignancies, including a prominent role in MALT lymphoma and activated B-cell type DLBCL [122]. However, there is rationale to expect MALT inhibition to have a potential effect in most lymphomas dependent more generally upon the BCR and NF-κB pathways, including the indolent lymphomas. Furthermore, as MALT1 is directly proximal to IKK/NF-κB, inhibition may overcome some of the resistance mechanisms that occur with inhibitors of more upstream components [123]. 

Three main classes of MALT1 inhibitors are in development: allosteric, covalent and protein degraders [121,123]. Pre-clinical studies have demonstrated in vivo and in vitro suppression of the growth of the ABC-DLBCL cell [123,124,125]. JNJ-67856633 [126] is an allosteric MALT1 inhibitor currently undergoing clinical studies in B-NHL and CLL, either as monotherapy (NCT67856633) or in combination (NCT04876092 with ibrutinib; NCT04657224 with JNJ-64264681, a second-generation BTKi). 

#### 2.4.7. IKK/NF-κB

In addition to the abovementioned agents targeting the upstream components of the NF-κB pathway (e.g., BTK, MALT and PI3K inhibitors), other inhibitors of IKK and NF-κB are also in development. These include agents targeting the NEDD8-activating enzyme, which regulates polyubiquitination of key intermediaries, such as IκBα, normally involved as a negative regulator of NF-KB transcription [127,128]. Pre-clinical studies suggest activity in DLBCL and MCL [128] with a phase 1 study of pevonedistat demonstrating a tolerable safety profile but limited responses in 17 patients with myeloma and 27 with lymphoma [129]. The combination with ibrutinib demonstrated improved response rates, particularly in MCL (ORR 100% and CR 50%), although patient numbers were small and additional efficacy over ibrutinib needs to be confirmed [130]. 

#### 2.4.8. JAK/STAT

The JAK/STAT signalling pathway provides a critical messaging conduit between the cellular membrane and the nucleus. It consists of two families, JAK and STAT, each with four and seven members, respectively. A host of external cytokines, chemokines and other ligands interacting with surface receptors have their effects mediated through this transduction cascade [131]. Aberrant activation, predominantly in the form of gain-of-function mutations, is associated with haematologic malignancies, including the myeloproliferative neoplasms and some forms of leukaemia and lymphoma [132]. Important cross-talk occurs with other signalling pathways, such as NF-kB, PI3/AKT/mTOR and NOTCH [132]. 

JAK inhibitor development has concentrated on rheumatologic disorders and select haematologic neoplasms. Cerdulatinib, the aforementioned SYK/JAK1/3 inhibitor, was tested in a phase 1 study in 43 patients with R/R B-NHL, including 13 with FL [133]. Dose-limiting toxicities included nausea, fatigue and lipase elevations. Two patients with FL experienced CR and 1 PR. In a related phase 2 study utilising cerdulatinib as monotherapy or in combination with rituximab, ORR rates were 53% and 77% with CR in 23.5% and 23%, respectively, in a cohort of patients with R/R FL (n = 60) [134]. 

#### 2.4.9. NOTCH1

Notch signalling controls several essential cellular processes, including determination of cell fate, self-renewal and tissue homeostasis, and is conserved across all metazoan species [135]. The notch pathway consists of the NOTCH receptors (NOTCH 1–4), key ligands from the Delta-like and Jagged families and associated coactivators. In a process incorporating the action of γ-secretase, the activation of NOTCH receptors results in the release of an intracellular domain, transportation to the nucleus and target gene transcription, before ultimate ubiquitin ligase degradation [136,137]. NOTCH1 overactivation was first identified in the pathogenesis of T-ALL, but Notch dysregulation has since been demonstrated in a broader range of lymphoid malignancies [138,139]. For instance, NOTCH2 gain-of-function mutations are frequent in SMZL, occurring in 20–25% of cases, and are associated with adverse clinical outcomes [140]. 

Notch targeting therapies include direct inhibitors, γ-secretase inhibitors, decoy molecules and monoclonal antibodies against Notch proteins. However, to date, most agents have been limited by clinical toxicity, most specifically gastrointestinal adverse events [136]. Limited data exist for use in the indolent lymphomas; however, given the frequency of Notch mutations and pathway dysregulation in lymphoid malignancies, a future role remains possible, including as a combination partner.

## 3. Targeted Immunotherapeutics

Targeted immunotherapeutics have expanded dramatically over the last two decades and the full spectrum is beyond the scope of this review. Most promising amongst the novel approaches in this domain are chimeric antigen receptor T-cells (CAR-T), CAR-NK cells and bispecific T-cell recruiting antibodies, all with emerging evidence for use in the indolent lymphomas, particularly FL [141,142,143,144,145]. Expanded investigation into WM/LPL, MZL and other indolent forms is awaited. 

This review will focus on monoclonal antibodies, including antibody-drug conjugates and the bispecific antibodies, CAR T cells, and will briefly review key highlights of the literature on lenalidomide.

### 3.1. Monoclonal Antibodies

Monoclonal antibodies (mAb) bind to surface receptors and target malignant cells for destruction through both immune clearance and intrinsic apoptotic pathways. In addition to naked antibodies, mAbs can be attached to other effector molecules, such as radioactive isotopes or chemotherapeutics.

#### 3.1.1. Anti-CD20

CD20 is present on mature B-cells and most malignant B-lymphocytes and was the target for the first approved monoclonal antibody, rituximab, for use in malignancy [146]. Rituximab is a type I mAb targeting CD20 that induces both complement dependent cytotoxicity (CDC) and antibody-dependent cellular cytotoxicity (ADCC). Second-generation anti-CD20 antibodies have been developed that differ in their potency and specificity for mechanisms of tumour cell death. Ofatumumab demonstrates increased C1q binding leading to a greater role for CDC in its efficacy profile. Obinutuzumab is a type II mAb with a glycoengineered FC portion, which provides higher affinity for the FcγR and improved ADCC functions. 

Anti-CD20 antibodies have been used in B-NHL for over two decades and have an established role, demonstrating efficacy both as monotherapy and in combination [147,148,149,150,151].

#### 3.1.2. Anti-CD19

CD19 is an immunoglobulin-superfamily surface protein involved in the enhancement of BCR signalling and is homogenously expressed on B-cells. Expression occurs from early stages of development through to near-terminal differentiation and is preserved in most B-cell lymphomas [152], making it an attractive target for naked, bispecific or conjugated antibodies and cellular immunotherapies. 

Two anti-CD19 mAbs have demonstrated promise in the treatment of indolent lymphoma. The first, inebilizumab, is a humanised mAb tested as monotherapy in a number of phase 1/2 studies in R/R FL, demonstrating ORR up to 82% and CR of 27% across various dosing cohorts [153,154]. Despite this promise, inebilizumab with chemotherapy did not demonstrate any benefit over combinations using standard anti-CD20 mAbs. The second, tafasitamab, is a humanised mAb with an engineered Fc domain that enhances ADCC activity and increases potency [155]. Responses were observed in 10 of 34 patients with R/R FL treated with tafasitamab monotherapy, lasting ≥12 months in four [156]. The most frequent treatment-emergent AE was neutropenia, occurring in 12% of patients. Of interest, tafasitamab gained accelerated FDA approval in R/R DLBCL on the basis of a phase 2 study in combination with lenalidomide (L-MIND), where ORR was 60%, including CRR of 43% [157]. The median DOR was 21.7 months and median OS was not reached at a median follow up of 13.2 months. Similar studies relevant to indolent lymphoma are ongoing, including in combination with lenalidomide-rituximab in FL/WM (NCT04680052) or acalabrutinib in MZL (NCT04646395). 

### 3.2. Antibody Drug Conjugates

Antibody drug conjugates (ADCs) comprise mAbs connected by a linker structure to a cytotoxic payload [158]. Binding of the ADC to the target ligand on malignant cell surfaces leads to internalisation and release of the payload compound with resultant cell death. Commonly used cytotoxic agents include tubulin inhibitors and DNA interacting agents [158]. ADCs targeting various B-cell markers relevant to indolent lymphomas are in clinical use and development. 

Polatuzumab vedotin is a CD79b ADC linked with the tubule poison monomethyl auristatin-E (MMAE) and has demonstrated efficacy in R/R FL. In the phase 2 ROMULUS study, R-pola was administered to 20 patients with FL demonstrating ORR of 70% with 45% achieving CR [159]. A combination of pola-obinutuzumab-lenalidomide has also been explored in 56 patients with R/R FL, again demonstrating significant responses, with ORR Of 76% and CR of 63% [160]. Infective complications with this combination were observed, including 9% febrile neutropenia and one septic death. A related combination of pola-obinutuzumab-venetoclax is also active (CR rate 57%) [79]. Various other combination studies are underway, including with bispecific antibodies (NCT03671018 and NCT03533283) [161,162], checkpoint inhibitors (NCT02729896), small molecule inhibitors (NCT04739813) or chemotherapy (NCT03677141). 

Loncastuximab tesirine is an ADC directed at CD19 and conjugated to a DNA crosslinking pyrrolobenzodiazepine toxin. A phase 1 study resulted in FDA approval in R/R DLBCL, and included results for 14 patients with FL and six with MZL [163]. The overall response rate was 45.6% in all histologies, with 78.6% in FL, which requires further validation due to small patient numbers. Improved anti-tumour activity has been demonstrated with the use of the dual ADCs, loncastuximab tesirine and polatuzumab vedotin, in xenograft models [164] and forms the basis of an upcoming study investigating the combination, alongside combinations with chemotherapy, lenalidomide or umbralisib (NCT04970901). 

The anti-CD22 ADC inotuzumab ozogamicin is a further agent with documented efficacy in FL. In combination with rituximab, an ORR of 87% with 2-year PFS of 68% was observed in 39 patients with R/R FL [165]. A further phase 2 study combined with R-CVP, including 27 patients with indolent lymphoma, demonstrated an ORR of 100% [166]. Haematologic toxicity was common, including thrombocytopenia requiring treatment cessation in 21%. 

A number of other ADCs have been developed, including additional agents targeting CD19, CD22 and CD79B as well as agents directed at CD20, CD25 and CD37, amongst others [158]. Further evidence is awaited on the role of these agents in indolent lymphoma. 

### 3.3. Bispecific T-Cell Recruiting Antibodies

Bispecific T-cell recruiting therapies simultaneously bind to surface proteins on endogenous T-cells and malignant B-cells, leading to directed activation of T-cell signalling, cytotoxic effector functions and malignant cell destruction. T-cell activation is predominantly achieved by targeting the T-cell receptor (TCR) sub-component, CD3, the binding of which can result in T-cell activation irrespective of TCR specificity or major histocompatibility complex class restriction [167]. Common B-cell targets include CD20 and CD19 due to their ubiquity and relatively preserved expression on malignant B-cells. 

There are a multitude of bispecific agents currently in development which each differ in their structure, mode of administration and physiochemical properties, including valency of binding sites and flexibility of linker proteins, which provide individual agents with unique pharmacodynamic and pharmacokinetic profiles [168]. The key toxicity associated with the bispecific agents is cytokine release syndrome, observed in up to 70% of treated patients [167]. In this syndrome, activated T-cells produce an excess of cytokines that initiate a cascade effect, including the recruitment, activation and secretion of mediators by myeloid and other immune cells, resulting in a potentially fatal systemic inflammatory state [169]. A range of mitigation strategies are in use to limit CRS with bispecific antibodies, such as step-up dosing, anti-CD20 pre-treatment and prophylactic steroid administration, which have significantly reduced the incidence of high-grade events [142,170,171]. Neurotoxicity, or immune effector cell neurotoxicity syndrome (ICANS), is a related disorder that often occurs concurrently with CRS, and is again driven by inflammatory cytokines and endothelial activation resulting in blood–brain barrier dysfunction [172]. Observed most prominently with CD19 targeting blinatumomab, neurotoxicity has been infrequent with newer generation CD20-directed agents [167]. This may in part relate to the presence of CD19 on supportive cells within the blood–brain barrier [173], although the experience of neurotoxicity with cellular immunotherapies directed at alternative targets, including CD20, suggests that target antigen alone cannot account for this difference [172].

The most advanced agents relevant to the indolent lymphomas each utilise a CD20/CD3 binding strategy with the majority of agents employing a 1:1 CD20:CD3 configuration (Table 4). These include mosunetuzumab, a full-length, fully humanised IgG1-based bispecific antibody tested as monotherapy in R/R FL. In a single-arm, phase 1 study of 68 patients with indolent lymphoma, the ORR was 66.2% with a CR rate of 48.5% [174]. Updated results from a phase 2 expansion cohort of 90 patients with FL and a median of three prior therapies demonstrated independently assessed ORR of 80%, including 60% CR, and PFS of 17.9 months [175]. Key toxicities included cytokine release syndrome (44%), predominantly grade 1–2 (42%), with low rates of neurotoxicity (4.4%) and AE-related treatment discontinuation (2.2%). Odronextamab demonstrated similar efficacy and toxicity in an early phase study including 37 patients with FL and smaller numbers with MZL (n = 6) and other indolent NHLs (n = 2) [176]. Epcoritamab primarily differs in its subcutaneous administration and again has demonstrated promising efficacy in a smaller cohort of 12 patients with R/R FL [177]. Importantly, no high-grade CRS was observed and there were no treatment discontinuations due to adverse events with this agent.

Glofitamab is a full-length bispecific utilising an IgG1-like fragment crystallisable (Fc) region with a unique 2:1 Fab arrangement, consisting of two CD20 domains and one CD3 domain [178]. This increase in CD20 valency creates increased potency and tighter immune-synapse formation, which provides for activity even in the presence of the concurrent anti-CD20 antibody [179,180]. In a phase 1b study of glofitamab including 72 patients with R/R FL, the ORR was 81% and 100% in monotherapy (n = 53) or in combination with obinutuzumab (n = 19), respectively, including CR rates of 70% and 74%, respectively [181]. At doses of ≥10 mg, the complete response rate in FL of 58.6% compares favourably to CR rates achieved in DLBCL and tFL of 42.15% and 64.3%, respectively [142]. At a median follow up of 8.6 months (range 0–37 months), the median duration of CR was not reached, with 83% of patients maintaining CR at last follow up, suggestive of significant durability [182]. 

Recent updated results, particularly with respect to response duration with mosunetuzumab and glofitamab, provides a strong indication that bispecific antibodies represent an effective treatment option in multiplied pre-treated indolent FL, which may translate to other indolent lymphomas. Relevant considerations in the establishment of their use will include the fixed treatment duration of most agents (with exception of epcoritamab) compared to indefinite treatment, in-depth analysis of the adverse event profile compared to targeted or CAR cellular approaches and longer follow up to assess for any plateau in progression-free and overall survival. With registration of these agents in indolent and aggressive lymphoma appearing imminent, research activities are now focused on combination therapies, including the utilisation of bispecific antibodies in frontline therapy. Combination studies with chemotherapy, targeted agents and additional T-cell costimulatory antibodies are in progress [167,168,178]. 

### 3.4. CAR T-Cells

CAR T-cells are a genetically modified cellular immunotherapy characterised by the ex vivo insertion of an engineered synthetic receptor onto the surface of T-cells, designed to promote recognition and subsequent destruction of malignant cells. There have been multiple iterations in CAR design that have led to the currently spectrum of licensed and investigational products, a process well described elsewhere [183,184]. Most modern CARs comprise an extra-cellular antigen-binding domain, a hinge domain and intracellular signalling domains, the latter importantly incorporating co-stimulatory motifs essential for effective T-cell activity [184]. 

Similar to the bispecific antibodies, significant clinical toxicity is well documented with CAR T-cell use, particularly CRS and ICANS. Rates of these toxicities differ according to product and lymphoma type, as well as patient factors, with emerging evidence that an elevated baseline inflammatory state and disease bulk both predict for poorer outcomes [172]. Long-term effects of CAR T-cells can also include cytopenias, often delayed, and hypogammaglobulinaemia due to persistent B-cell aplasia [185]. 

CD19-directed CAR T-cells are widely utilised in the treatment of DLBCL, with approval based primarily on pivotal phase 1/2 studies [186,187,188]. Similarly, axicabtagene ciloleucel has recently gained accelerated FDA approval for use in R/R FL on the basis of the single-arm phase 2 ZUMA-5 study (Table 5) [189]. In total, 153 patients with R/R FL or MZL after at least two prior lines or therapy were enrolled, with 148 receiving cellular infusion. Eighty-four patients with FL and 20 with MZL were evaluable for efficacy assessment, demonstrating an ORR of 92%, CRR of 74% and 18 month PFS of 64.8%. Grade ≥3 CRS occurred in 7% and grade ≥3 neurotoxicity in 19% of patients, with four deaths (3%) due to adverse events. 

Tisagenlecleucel, a second CD19 CAR T, has also recently been investigated in the phase 2 ELARA study and is awaiting expedited FDA review (Table 5) [145]. Results from 97 infused patients with R/R FL demonstrated similar ORR of 86.2%, CRR of 69.1% with lower rates of high-grade CRS (0%) and neurotoxicity (1%) in keeping with tisagenlecleucel’s more favourable safety profile in DLBCL. Although no grade 3–4 CRS events were observed, grade 1–2 CRS was observed in 48.5% overall and was more frequent in patients with bulky disease (57%).

Further data are awaited for both of these agents and other CAR products in the indolent lymphomas, which will be crucial in informing how best to incorporate these novel cellular immunotherapies into existing treatment algorithms [190]. Both Axicabtagene and tisagenlecleucel demonstrate significant efficacy, including in patients with multiple treated FL and high-risk features, such as POD24, and may form an important therapeutic option for such patients with high unmet clinical need. However, both short and long-term toxicities must be taken into account. Combined with the significant resource burden and limited access to CAR products generally, this may result in off-the-shelf treatments, such as bispecific antibodies, being a more viable treatment option for the majority of patients. 

Finally, a number of novel cellular immunotherapy approaches are being explored, including additional modifications to CAR T-cells, such as ‘safety-switches’, or the use of alternative effector cells with more favourable safety profiles, which may further alter the landscape [191,192]. For instance, allogeneic CAR-NK cells have the advantage of natural anti-tumour properties, avoidance of the need for autologous ex vivo manufacture delay, and more limited expansion potential, which may obviate much of the potential for CRS and neurotoxicity [193]. However, persistence has been the traditional barrier to development of NK therapies [194]. A phase 1/2 study of CD19 directed CAR-NK cells in 11 patients with B-NHL (5 CLL, 2 DLBCL, 3 tFL and 1 FL) attempted to address this problem by providing a further Il-15 signal within the product. An ORR of 73% with no CRS, neurotoxicity or graft-versus-host disease was observed [195]. Despite this promising advance over prior NK products in the clinic, five of the eight responding patients in that study received post-remission therapy and we cannot conclude that the issue of NK-cell persistence is resolved. 

### 3.5. Lenalidomide

Lenalidomide is a T-cell and NK-cell activating agent with multiple immunomodulatory effects. Lenalidomide is thought to primarily act through binding to the cereblon E3 ubiquitin ligase complex resulting in the degradation of transcription factors and resultant direct apoptotic death [196]. Pre-clinical studies have demonstrated a synergistic effect when combined with the ADCC mechanism of mAbs [197,198], which has borne out in human clinical trials. Further therapeutic effects of lenalidomide include downregulation of checkpoint molecules on malignant cells and cytokine-mediated alteration of the tumour microenvironment, the latter forming an essential component in the maintenance of many lymphoproliferative disorders [199,200]. Changes include inhibition of microvascularisation, reduction in tumour-associated macrophages, improvement in T-cell motility and repair of T- and NK-synapse formation [200,201].

The combination of lenalidomide and rituximab (‘R^2^’) has demonstrated efficacy in both upfront FL and RR FL and WM [202]. In the phase 3 RELEVANCE study R^2^ was compared to the physician’s choice in the frontline treatment of advanced stage FL, with co-primary endpoints of CR rate and PFS [203]. The study failed to demonstrate superiority of the chemo-free regimen, with similar CR rates (48% vs. 53%) and overlapping PFS curves. Although not formally designed as a non-inferiority study, the large sample size (1030 patients), narrow PFS confidence intervals and median follow up of 38 months, many have reasonably interpreted the study as establishing equivalence of R^2^ to R-chemotherapy in frontline FL. While adverse events of any grade were lower in the R^2^ group (66% vs. 89%), it must be noted that dose interruptions (59% vs. 35%), dose modifications (36% vs. 14%) and early discontinuations (11% vs. 3%) due to treatment adverse events were more common in the R^2^ group. Adverse events higher in the R^2^ included cutaneous reactions (43% vs. 24%) and diarrhoea (37% vs. 19%), while peripheral neuropathy (7% vs. 16%) and febrile neutropenia (2% vs. 7%) were more common with chemotherapy. Thromboembolic events were equal between groups (3% each), although prophylaxis was recommended for patients on the R^2^ arm.

In the phase 3 AUGMENT study, an R^2^ regimen was compared against placebo plus rituximab in the setting of R/R but rituximab-sensitive FL (n = 295) and MZL (n = 63) [204]. Overall, the median PFS was significantly longer in the R^2^ arm (39.4 months vs. 14.1 months), which was similar to the results when restricted to FL (39.4 months vs. 13.9 months). Although not powered for this purpose, sub-group analysis in WM did not show a convincing benefit; however, based on the success of the primary end-point FDA approval was granted for both indications. There are a number of limitations to consider, such as the different duration of therapy between arms, a weak comparator arm of single-agent rituximab and observed toxicities, which included grade 3–4 neutropenia in 50%, transient tumour-flare in 10% and the risk for thromboembolism [202]. 

The combination of lenalidomide and rituximab has also been tested in WM, with a phase 2 study of 16 patients demonstrating ORR of 50% but high rates of unexpected clinically significant anaemia (13 of 16 patients) [205]. Single agent lenalidomide in a subsequent study reduced rates of anaemia, but response rates were also lower, with ORR of 29% [206]. 

## 4. Conclusions

Recent significant advances in targeted therapies for indolent lymphoma has resulted in a plethora of new and emerging treatment options extending far beyond the traditional cytotoxic chemotherapy paradigm. Many of these agents have the potential to improve outcomes by rationally targeting features of malignant B-cells that make them uniquely sensitive. While this can in some cases reduce treatment side effects, this is not always the case, and therefore systematic and stepwise development of the targeted therapy armamentarium is essential. This review has highlighted some of the key approved agents as well as novel areas undergoing further investigation and foreshadows an exciting future in the treatment of indolent lymphomas. 

## Figures and Tables

**Table 1:** Summary of key BTK inhibitors.

Disease	Setting	Agent(s) and Dosing	Trial (Sample Size)	Efficacy	Relevant Toxicities	Comments	FDA Approval
**Follicular** **Lymphoma**	R/R FL	Ibrutinib 560 mg daily	Single arm Ph 2 (40 pts) [21]	ORR 37.5%, median PFS 14 Mo	Arrhythmia 2.5% Gr3+ bleeding 5% (fatal 2.5%) Gr3+ infection (7.5%)	No response if *CARD11mut*	No
	R/R FL	Ibrutinib 560 mg daily	Single arm Ph 2 (110 pts) [20]	ORR 20.9%, median PFS 4.6 Mo	Arrhythmia 3% Major haemorrhage 4% Gr3+ infection 7.3% (1 fatal)	Low ORR, but in responders DoR 19.4 Mo	No
**Marginal Zone** **Lymphoma**	R/R MZL	Ibrutinib 560 mg daily	Single arm Ph 2 (63 pts) [23]	ORR 58%, median DoR 27.6 Mo, median PFS 15.7 Mo	Arrhythmia 8% Gr3+ bleeding 3% (1 fatal^#^) Gr3+ infection 22% (1 fatal *)	Better outcomes with *MyD88mut*	Yes
	R/R MZL	Zanubrutinib 160 mg bd	Single arm Ph 2 (68 pts) [24]	ORR 74%, 62% PFS @ 12 Mo	Arrhythmia 1.5% Gr3+ bleeding NR Gr3+ infection NR	Short follow up	No
**Lymphoplasmacytic Lymphoma/Waldenstrom** **Macroglobulinaemia**	R/R WM	Ibrutinib 420 mg daily	Single arm Ph 2 (63 pts) [25]	ORR 90.5%, 54% PFS @ 5 years	Arrhythmia 13% Gr3+ bleeding NR Gr3+ infection 6%	Inferior outcomes if *MyD88wt* or *CXCR4mut*	Yes, alone or in combination with rituximab
	Frontline or R/R WM (INNOVATE)	Ibrutinib 420 mg daily and Ritux vs. Placebo and Ritux	Phase 3 with cross-over post PD (75 pts per arm) [26]	ORR 92% vs. 47% In R/R 30 Mo PFS rate 82% vs. 22% (P < 0.0001)	Ibrutinib-Ritux: Arrythmia 15% Gr3+ bleeding 4% Gr3+ pneumonia 9% Placebo-Ritux: Arrhythmia 3% Gr3+ bleeding 4% (1 fatal) Gr3+ pneumonia 3%	No overall survival difference. Ibr superior in all molecular subsets	Yes, alone or in combination with rituximab
	R/R WM	Acalabrutinib	Phase 2 (92 pts) [27]	ORR 93% 82% PFS @ 2 years	Arrhythmia 1% Gr3+ bleeding 3% (1 fatal) Gr3+ pneumonia 7%	Similar responses in *MyD88mut* and *MyD88wt*	No
	R/R WM (ASPEN)	Zanubrutinib 160 mg bd vs. Ibrutinib 420 mg daily	Phase 3 (~100 pts per arm) [28]	ORR 95% both arms 12 Mo PFS 90% vs. 87% (P = NS)	Zanubrutinib: Arrhythmia 2% Gr3+ bleeding 6% Gr3+ pneumonia 1% Ibrutinib: Arrhythmia 15% Gr3+ bleeding 9% Gr3+ infection 7%	Fewer treatment D/C with Zanu (4 vs. 9%). Less Afib (2 vs. 14%), but more neutropenia	No
	Frontline or R/R WM	Tirabrutinib	Dual cohort phase 2 (Treatment naïve 18 pts, R/R 9 pts) [29]	ORR 94% TN and 100% R/R 24 Mo PFS 94% TN and 89% R/R	Arrhythmia 7% Gr3+ bleeding 0% Gr3+ infection 3.7%	Small numbers in both cohorts	No (registered in Japan for frontline and R/R WM)
	R/R WM	Orelabrutinib	Single arm Ph 2 (47 pts) [30]	ORR 87% 12 Mo PFS 88%	Arrhythmia 0% Gr3+ bleeding 0% Gr3+ infection 4.3%	Higher rates of major response with *MYD88mut CXCR4wt* Short follow up (median 11 months)	No

Legend: WM, Waldenstrom Macroglobulinaemia; R/R, relapsed-refractory; ORR, overall response rate; TN, treatment-naïve; DoR, duration or response; PFS, progression free survival; FL, follicular lymphoma; MZL, marginal zone lymphoma; D/C, discontinuation; NR, not reported; #, unlikely related, *, possibly related.

**Table 2:** PI3K inhibitors—FL and MZL subsets.

Agent (Dose and Route)	Target(s)	Trial (Sample Size)	Efficacy	Gr3+ AE’s	Drug D/C Rate Due to AE’s	FDA Approval
**Idelalisib[50,51]** 150 mg oral bd	PI3Kδ	Ph 2 (72 FL)	Follicular lymphoma subset ORR 56% (14% CR) Median PFS 11 Mo	Diarrhea/colitis (16%), pneumonia (7%), hepatic (13%), neutropenia (27%)	20%	Yes, after 2 lines
**Duvelisib[52]** 25 mg oral bd	PI3Kγ and PI3Kδ	Ph 2 (83 FL)	Follicular lymphoma subset ORR 42% (1% CR) Median PFS 9.5 Mo	Diarrhea/colitis (20%), pneumonia (5.4%), hepatic (5.4), febrile neutropenia (9.3%)	24%	Yes, after 2 lines
**Copanlisib[53]** 60 mg 1hr IV infusion, D1, 8, 15 q 28d	PI3Kα and PI3Kγ	Ph 2 (104 FL)	Follicular lymphoma subset ORR 59% (20% CR) Median PFS 12.5 Mo	Hyperglycemia (40%), hypertension (24%), diarrhea (9%), pneumonia (11%)	26.8%	Yes, after 2 lines
**Umbralisib[54]** 800 mg oral daily	PI3Kδ and casein kinase-1ε	Ph 2 (117 FL, 69 MZL)	Follicular lymphoma subset ORR 45% (5% CR) Median PFS 10.6 Mo MZL subset ORR 49% (16% CR) 2 year PFS 51%	Diarrhea (10%), hepatic (7%), neutropenia (12%)	15%	Yes, after 3 lines

Legend: ORR, overall response rate; PFS, progression free survival; FL, follicular lymphoma; MZL, marginal zone lymphoma.

**Table 3:** Venetoclax in indolent lymphomas.

	Regimen	Trial (MZL Sample Size)	Efficacy	Gr3+ AE’s	Comments
**Follicular Lymphoma** **[70,71]**	Single agent 200–1200 mg oral daily	Ph 1/2 (29 FL)	ORR 38% (17% CR) Median PFS 10.8 Mo	47% overall GI tolerance dose limiting 17% Gr3+ neutropenia	Median DoR for CR = 37.6 Mo
**Follicular Lymphoma** **[68]**	800 mg/d plus rituximab 800 mg/d plus BR	Ph 2 52 (plus ritux) 51 (plus BR)	ORR 35% (17% CR) ORR 84% (75% CR)	50% (6% deaths) 92% (2% deaths, 6% PJP)	Addition of Ven did not improve outcomes with BR alone
**Marginal Zone Lymphoma** **[70,71]**	Single agent 200–1200 mg oral daily	Ph 1/2 (3 MZL)	ORR 67% (all PR)	---	
**Waldenström Macroglobulinemia** **[69]**	Single agent 800 mg/d oral 2 years	Ph 2 (30)	ORR 87% 74% major response rate PFS 83% @ 12 months	21% neutropenia 3% infections	Active if prior Ibrutinib or *CXCR4mut*

Legend: ORR, overall response rate; PFS, progression free survival; FL, follicular lymphoma; MZL, marginal zone lymphoma; DoR, duration of response; BR, bendamustine plus rituximab; PR, partial response; CR, complete response; Ritux, rituximab.

**Table 4:** Emerging bispecific antibodies in follicular lymphoma.

Agent (Dose and Route)	Targets	Trial (Sample Size)	Efficacy	Significant AE’s	Drug D/C Rate Due to AE’s	FDA Approval
**Mosunetuzumab[174,175]** Step-up dosing IV administration	1:1 CD20/CD3	FL (90 pts)	ORR 80%, CRR 60% Median PFS 17.9 mo Median DOR not reached (median f/u 18.3 mo)	CRS 44% (Grade 3 1%, Grade 4 1%) Neurotoxicity 4.4%	2.2%	Yes
**Odronextamab[176]** Step-up dosing IV administration	1:1 CD20/CD3	FL (28 pts)	ORR 92.9%, CRR 75% Median DOR 7.7 mo	CRS 62.2% (Grade 3 6.3%, Grade 4 0.8%) Neurotoxicity 2.3%	5.5%	No
**Epcoritamab[177]** Step-up dosing (priming and intermediate doses) SC administration	1:1 CD20/CD3	FL (10 pts evaluable)	ORR 90%, CRR 50% DOR and PFS not reported	CRS (all 68 pts, including DLBCL) 59% (all Grade 1–2) Neurotoxicity 6%	0%	No
**Glofitamab[181]** Step-up dosing Monotherapy or in combination with obinutuzumab IV administration	2:1 CD20/CD3	FL (72 pts: 53 mono, 19 combo)	Monotherapy ORR 81%, CRR 70% Combination ORR 100%, CRR 74% Median DOCR not reached (median f/u 8.6 mo) [182]	CRS 66% mono, 79% combo (Grade 3 1 pt mono only) Neurotoxicity 36% (0% ICANS)	0% mono 5.3% combo	No

Legend: IV, intravenous; SC, subcutaneous; ORR, overall response rate; PFS, progression free survival; FL, follicular lymphoma; DOR, duration of response; DOCR, duration of complete response; CRR, complete response rate; CRS, cytokine release syndrome; D/C, discontinuation.

**Table 5:** CAR T-cell products in FL.

Agent	Target	Number Infused	Patient Characteristics	Efficacy	Significant AE’s	FDA Approval
Axicabtagene ciloleucel [189]	CD19	148 (124 FL, 24 MZL)	Stage III-IV 86% Bulky disease 50% Median prior lines 3 (range 2–4) POD24 55%	ORR 92% CRR 74% 18 mo PFS 64.8%	Grade ≥ 3 CRS 7% Grade 3 ≥ ICANS 19%	Yes
Tisagenlecleucel [145]	CD19	97 FL	Stage III-IV 85.6% Bulky disease 63.9% Median prior lines 4 (range 2–13) POD24 62.9%	ORR 86.2% CRR 69.1% 12 mo PFS 67%	Grade ≥ 3 CRS 0% Grade ≥ 3 ICANS 1%	Awaiting review

Legend: POD24, progression of disease ≤24 mo after anti-CD20 monoclonal antibody treatment; FL, follicular lymphoma; MZL, marginal zone lymphoma; ORR, overall response rate; CRR, complete response rate; PFS, progression free survival; CRS, cytokine release syndrome; ICANS, immune effector cell neurotoxicity syndrome.
